# Carotid intima‐media thickness predicts carotid plaque development: Meta‐analysis of seven studies involving 9341 participants

**DOI:** 10.1111/eci.13217

**Published:** 2020-03-17

**Authors:** Lena Tschiderer, Gerhard Klingenschmid, Lisa Seekircher, Peter Willeit

**Affiliations:** ^1^ Department of Neurology Medical University of Innsbruck Innsbruck Austria; ^2^ Department of Public Health and Primary Care University of Cambridge Cambridge UK

**Keywords:** carotid intima‐media thickness, carotid plaque, meta‐analysis, prospective studies

## Abstract

**Background:**

Carotid intima‐media thickness and carotid plaque are well‐established imaging markers used to capture different stages of the atherosclerotic disease process. We aimed to quantify to which extent carotid intima‐media thickness predicts incidence of first‐ever carotid plaque.

**Materials and methods:**

Two independent reviewers conducted a comprehensive literature search of PubMed and Web of Science. To be eligible for inclusion, prospective studies were required to involve participants free of carotid plaque at baseline and report on the association of baseline carotid intima‐media thickness with development of first‐ever carotid plaque. Study‐specific relative risks and 95% confidence intervals were collected and pooled using random‐effects meta‐analysis.

**Results:**

We identified seven relevant prospective studies involving a total of 9341 participants. Individuals were recruited between 1987 and 2012, average age at baseline was 54 years, and 63% were female. Studies reported on 1288 incident first‐ever carotid plaques, occurring over an average maximum follow‐up of 8.7 years. When individuals in the top fourth of baseline carotid intima‐media thickness distribution were compared with those in the bottom fourth, the pooled relative risk for incidence of first‐ever carotid plaque was 1.78 (95% confidence interval: 1.53‐2.07, *P* < .001, *I* ^2^ = 2.8%). The strength of association was not modified by mean baseline age, proportion of female participants, length of follow‐up, year of baseline, and geographical location of the studies.

**Conclusions:**

In general population studies, elevated baseline carotid intima‐media thickness is associated with incidence of carotid plaque in individuals free of carotid plaque at baseline.

## 1 INTRODUCTION

Atherosclerosis—a common cause of cardiovascular disease (CVD)—is characterised by alterations of the vessel walls, including accumulation of lipid deposits and initiation of inflammation leading to the development of arterial lesions.^1^ Quantifying atherosclerosis with imaging markers is an important tool in the prediction and prevention of CVD. Two imaging markers widely used to directly or indirectly assess atherosclerosis are carotid intima‐media thickness (cIMT) and carotid plaque—both measured with carotid ultrasound.

cIMT is usually measured by B‐mode ultrasound at different sites of the carotid arteries and is—as initially described in 1986 by Pignoli et al^2^—the distance between the intima‐lumen and adventitia‐media interface. Elevated cIMT has been implicated in development of CVD. It has been identified to be related to various traditional cardiovascular risk factors, abnormalities in other organ systems, and atherosclerosis in different arterial beds.^3^ In addition, it has been demonstrated to predict future CVD events.^4^

Carotid plaque can also be measured by applying B‐mode ultrasound techniques and can be assessed simultaneously to cIMT. Studies report on a variety of carotid plaque measures^5^ including carotid plaque occurrence,^6^ amount,^7^ thickness,^8^ area,^9^ density,^10^ plaque scores^11^ and 3‐dimensional assessments.^12^ Like cIMT, carotid plaque has been demonstrated to be associated with cardiovascular risk factors.^13^ Furthermore, a variety of studies have demonstrated that carotid plaque could be used as a prognostic marker for future cardiovascular events.^5^ It has also been demonstrated that carotid plaque outperforms cIMT in predicting future myocardial infarction.^14^

To which extent increased cIMT is related to future carotid plaque development is not entirely clear. On the one hand, cIMT and carotid plaque may represent distinct phenotypes of vascular remodelling.^15^, ^16^ This theory is underpinned by genetic studies that revealed distinct genes to be associated with cIMT and carotid plaque.^17^, ^18^ On the other hand, cIMT and carotid plaque are considered to represent different stages of atherogenesis characterised by continuous arterial wall growth. Following this theory would imply cIMT being a predictive measure for the initiation and development of new carotid plaque and would further endorse the relevance of cIMT as a tool to quantify atherosclerosis—and identify individuals at elevated risk for future CVD—at a very early stage. Numerous single studies have investigated the association between increased cIMT and carotid plaque. Cross‐sectional studies have examined the co‐existence of the two measures^19^, ^20^, ^21^, ^22^, ^23^, ^24^, ^25^, ^26^, ^27^ and identified strong to very strong associations. In addition, prospective studies have investigated associations between baseline cIMT and development of future carotid plaque^22^, ^23^, ^27^, ^28^, ^29^, ^30^, ^31^ leading to different conclusions and varying effect sizes. While the ARIC study,^23^ the EVA study,^30^ the San Daniele 2 Project^31^ and a combined analysis of the CMCS and the PRC‐USA study^29^ identified positive associations between baseline cIMT and development of carotid plaque, the Northern Manhattan Study,^22^ a study by Yang et al^27^ and a study by Bonithon‐Kopp et al^28^ did not confirm these associations when adjusting for potential confounders.

In order to provide an overview of current evidence and reliably quantify the relationship between elevated cIMT and development of carotid plaque, we performed a systematic literature review to identify studies that had reported on the association between baseline cIMT and incidence of first‐ever carotid plaque among individuals free of baseline carotid plaque and conducted a literature‐based meta‐analysis of individual study results.

## 2 MATERIALS AND METHODS

Reporting of this study conforms to the PRISMA statement (Table S1).^32^, ^33^ The review has not been registered a priori.

### 2.1 Literature search, study selection and data extraction

We systematically sought PubMed and Web of Science for prospective studies that were published until 18 October 2019 and reported on baseline cIMT and incidence of carotid plaque. We applied the following PubMed search terms: ("intima‐media thickness"[All Fields] OR "IMT"[All Fields] OR "intima media thickness"[All Fields]) AND "plaque"[All Fields] AND ("incident"[All Fields] OR "prospective"[All Fields]). In Web of Science, we searched for: TS=("intima‐media thickness" OR "IMT" OR "intima media thickness") AND TS=("plaque" AND ("incident" OR "prospective")). After eligible articles were identified, we additionally scanned references of these articles and searched for articles that have cited relevant articles (“snowball principle”) via Web of Science, further enhancing the coverage of our literature search.

To be included, studies were required to (a) have a prospective design, (b) have reported on the association of baseline cIMT with incidence of future carotid plaque (yes versus no) and (c) have reported this association in individuals free of carotid plaque at baseline. Two independent reviewers (LT and GK) systematically searched PubMed and Web of Science and identified relevant studies to be included in the current report. If studies qualified for inclusion, the reviewers independently extracted important information on the following characteristics: study location, year of baseline, duration of follow‐up, mean baseline age, percentage of female participants, mean and standard deviation (SD) of baseline cIMT, level of adjustment, number of participants, number of incident carotid plaques, comparison metric (per unit increase, per SD increase, top fourth versus bottom fourth and greater versus lower than a specific threshold level), reported relative risk (RR) and corresponding reported 95% confidence interval (CI). In addition, details about cIMT and carotid plaque assessment were extracted, including measurement side, wall and metric of cIMT, whether the same sonographer has conducted the ultrasound measurements, details about the ultrasound machine used, location of carotid plaque and criteria for presence of carotid plaque. If studies reported effect sizes with several levels of adjustment, the effect size of the most adjusted model was used. One study^22^ reported associations according to different measurement sites of baseline cIMT, and the effect size corresponding to cIMT at the common carotid artery (CCA) was used for analysis to enhance comparability to the other studies. Two studies^22^, ^30^ reported associations in subpopulations of individuals with and without carotid plaque at baseline, and—following our predefined inclusion criteria—we implemented RRs of participants having no carotid plaque at baseline.

### 2.2 Statistical analyses

We conducted analyses according to a predefined statistical analysis plan. The primary analysis focused on the association between baseline cIMT and development of first‐ever carotid plaque. Because studies reported effect estimates on different scales (per SD, per unit increase, top fourth versus bottom fourth and above versus below a specific threshold), we converted RRs and 95% CIs to reflect a comparison of the top versus bottom fourth of baseline cIMT distribution using methods described elsewhere,^34^, ^35^ assuming a normal distribution of cIMT at baseline and a log‐linear association with risk of incident carotid plaque. One study,^29^ consisting of two cohorts, did not report on the distribution parameters of cIMT in their manuscript, which is necessary for converting the RR, but we were able to retrieve the necessary information from other publications of these two cohorts.^34^, ^35^ We pooled study‐specific RRs using random‐effects meta‐analysis and additionally conducted fixed‐effect meta‐analysis for sensitivity analysis. The *I* ^2^ statistic was used to assess heterogeneity between studies.^36^ Subgroup analyses were conducted using meta‐regression across pre‐specified study‐level characteristics.^36^, ^37^ We evaluated whether publication bias was present by visually inspecting a funnel plot and applying Egger's asymmetry test.^38^ All statistical tests were two‐sided; *P*‐values ≤ .05 were deemed as statistically significant. Data were analysed using the statistical software Stata version 15.1 (StataCorp).

## 3 RESULTS

### 3.1 Eligible studies and general study‐level characteristics

**Figure 1:**
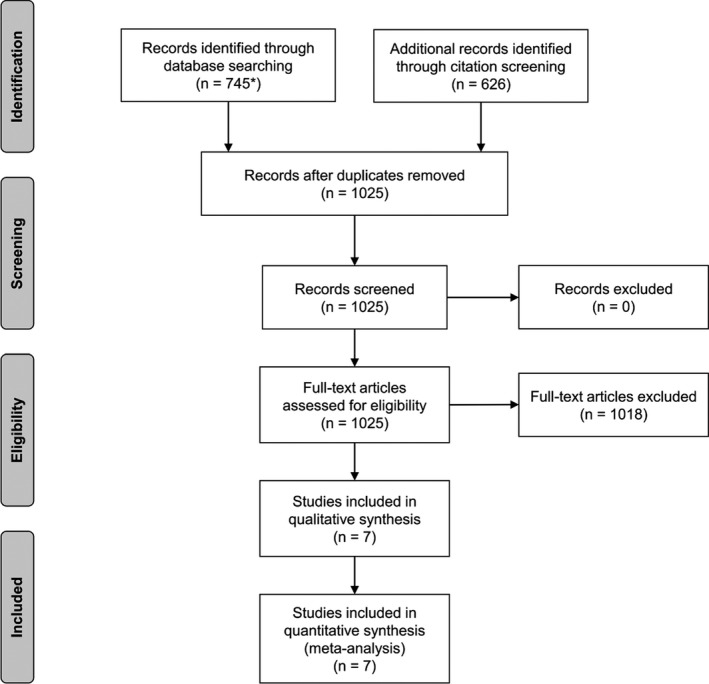
Study flow diagram. *387 articles were identified in PubMed and 358 in Web of Science

We identified 387 relevant articles from PubMed, 358 from Web of Science and another 626 through citation screening via Web of Science (Figure 1). After removing duplicate manuscripts, we assessed the remaining 1025 for eligibility, of which seven studies qualified for inclusion into the current analysis. Key characteristics of the included studies are summarised in Table 1. Three of the seven studies were based in Europe (two in France and one in Italy), two in the United States and two in China. All studies recruited individuals from the general population. Baseline years ranged from 1987 to 2012. The weighted maximum length of follow‐up was 8.7 years, weighted mean baseline age was 54.0 years, and 63% were female. In total, the studies involved 9341 participants free of carotid plaque at baseline and 1288 events of first‐ever carotid plaque (two studies^23^, ^31^ did not report on the number of incident carotid plaques).

**Table 1:** Study‐level characteristics of the included studies

Study	Country	Baseline year	Maximum follow‐up, years	Mean age, years	Female sex, %	No. of participants	No. of incident plaques
ARIC^23^	USA	1987‐89	11.7	53.0	62.0	4791	NR
Bonithon‐Kopp^28^	France	1988‐89	2.0	49.3	100.0	285	28
CMCS/ PRC‐USA^29^	China	2002	5.0	56.9	62.5	1590	780
EVA^30^	France	1991‐93	4.0	65.1	59.7	814	120
NOMAS‐INVEST^22^	USA	1993‐97	7.3	63.0	63.0	324	130
San Daniele 2^31^	Italy	1990	12.0	43.4	52.5	795	NR
Yang^27^	China	2011‐12	2.4^a^	51.5	72.2	742	230
Total		1987‐2012	8.7	54.0	63.0	9341	1288

Abbreviations: ARIC, Atherosclerosis Risk in Community; CMCS/ PRC‐USA, Chinese Multi‐Provincial Cohort Study/ People's Republic of China‐United States Collaborative Study in Cardiovascular and Cardiopulmonary Epidemiology; EVA, Étude du Vieillissement Artériel Study; NOMAS‐INVEST, Northern Manhattan Study‐Oral Infections and Vascular Disease Epidemiology Study; NR, not reported; San Daniele 2, San Daniele 2 Project.

a Mean follow‐up.

### 3.2 Assessment of cIMT and carotid plaque

Table 2 provides an overview of how studies have assessed cIMT. All seven studies reported baseline cIMT measured at the CCA. Definition of cIMT differed according to measurement side (left side versus right side), measurement wall (far wall versus near wall) and method of combination of single measurements (mean versus mean‐maximum). The ultrasound measurements were conducted by the same sonographer in three studies. All studies used different ultrasound machines with varying transducer frequencies from 7.5 to 13 MHz. Mean cIMT values at baseline ranged from 0.63 mm in the ARIC study to 0.91 mm in the NOMAS‐INVEST study. Overall, the pooled mean baseline cIMT was 0.73 mm (SD 0.18). Based on this overall distribution, values of baseline cIMT in the top fourth and the bottom fourth were estimated at > 0.85 mm and ≤ 0.61 mm.

**Table 2:** Assessment of carotid intima‐media thickness at the common carotid artery

Study	CCA side	CCA wall	Metric	Same sonographer	Type of ultrasound machine	Transducer frequency (MHz)	cIMT (mm) mean ± SD
ARIC^23^	Right	NR	NR	No	Biosound 2000 II sa	8	0.63 ± 0.14
Bonithon‐Kopp^28^	Right + left	Far	NR	Yes	NR	7.5	NR
CMCS/ PRC‐USA^29^	Right + left	Far	Mean‐maximum	No/NR^a^	ALOKA Prosound α10/ACUSON‐ASPEN 128^a^	7.5/7.5‐10^a^	0.78 ± 0.21
EVA^30^	Right + left	Far	Mean	No	Aloka SSD‐650	7.5	0.66 ± 0.11
NOMAS‐INVEST^22^	Right + left	Near + far	Mean‐maximum	Yes	GE LogIQ 700	9‐13	0.91 ± 0.11
San Daniele 2^31^	Right + left	Far	Mean‐maximum	Yes	4500/5500 HP	7.5	NR
Yang^27^	Right + left	Far	Mean	No	GE Vivid 7	8‐10	0.66 ± 0.08

Abbreviations: CCA, common carotid artery; cIMT, carotid intima‐media thickness; NR, not reported; SD, standard deviation.

a First item belongs to CMCS, second item belongs to PRC‐USA. Full study names are provided in the footnote of Table 1.

Table 3 provides an overview of how studies have assessed carotid plaque. Six studies obtained carotid plaque located at the CCA, the carotid bifurcation and the internal carotid artery, and one study assessed carotid plaque at the CCA and the carotid bifurcation only. All studies scanned carotid arteries for presence of plaque bilaterally. Studies reported different criteria to be fulfilled in order to determine presence of carotid plaque involving criteria related to focal shape (and appropriate conditions), increased cIMT (and a corresponding threshold of cIMT) and texture of the arterial wall.

**Table 3:** Carotid plaque assessment

Study	Plaque location	Involved criteria for plaque assessment^a^		
		Focal shape; condition	cIMT; threshold (mm)	Wall texture; condition
ARIC^23^	Right + left CCA + BIF + ICA	Yes; protrusion into lumen, loss of alignment, rough boundary	Yes; >1.5	Yes; brighter echoes than adjacent boundaries
Bonithon‐Kopp^28^	Right + left CCA + BIF	Yes; ≥1.75 mm^c^	No	No
CMCS/ PRC‐USA^29^	Right + left CCA + BIF + ICA	Yes; ≥0.5 mm^b^	Yes; ≥1.3	No
EVA^30^	Right + left CCA + BIF + ICA	Yes; ≥1 mm^c^	No	No
NOMAS‐INVEST^22^	Right + left CCA + BIF + ICA	Yes; >50% than surrounding	No	No
San Daniele 2^31^	Right + left CCA + BIF + ICA	Yes; ≥0.5 mm^b^ or ≥ 50% than surrounding	Yes; ≥1.5	No
Yang^27^	Right + left CCA + BIF + ICA	Yes; ≥0.5 mm^b^ or ≥ 50% than surrounding	Yes; >1.5	No

Abbreviations: BIF, carotid bifurcation; CCA, common carotid artery; cIMT, carotid intima‐media thickness; ICA, internal carotid artery. Full study names are provided in the footnote of Table 1.

a In the ARIC study, individuals had to meet two of the criteria; in the other studies, individuals had to meet one of the criteria.

b Thickness of structure encroaching into the arterial lumen.

c Distance between the media‐adventitia interface and the internal side of the plaque.

### 3.3 Association of baseline cIMT with incidence of first‐ever carotid plaque

**Figure 2:**
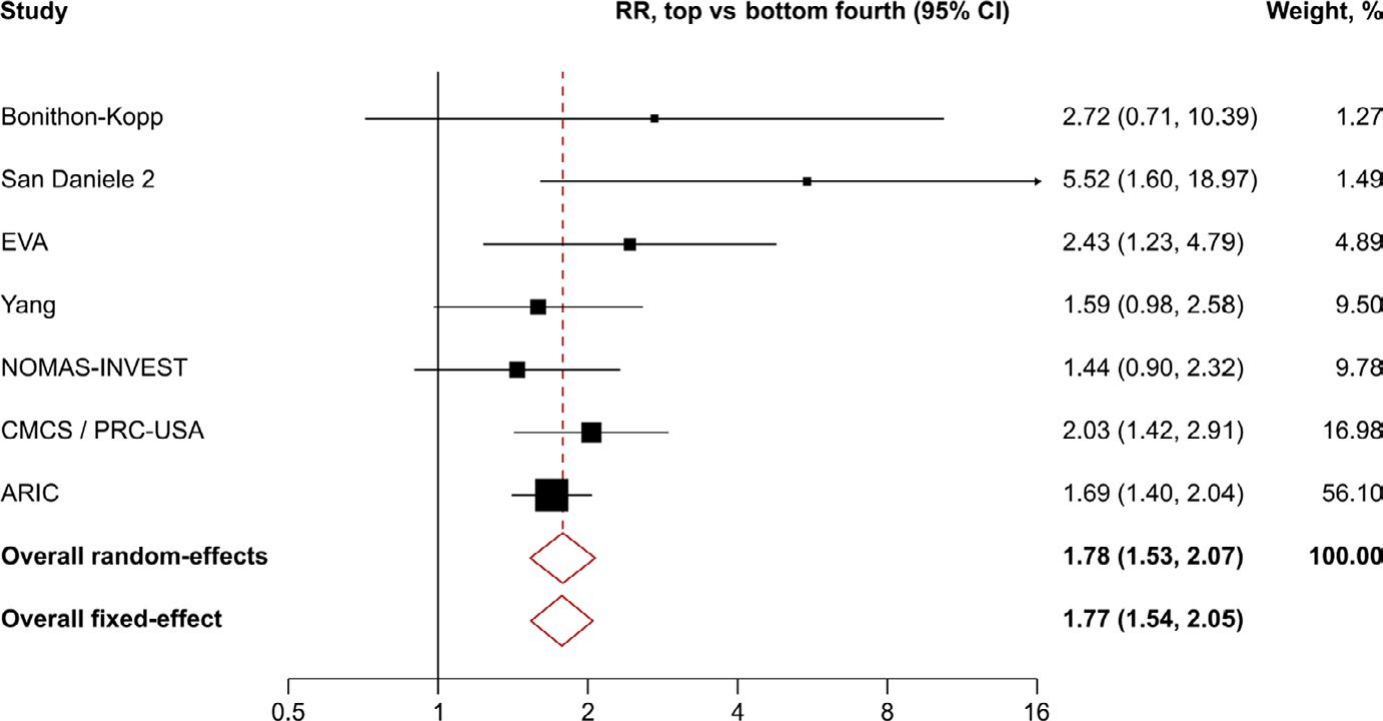
Overall association of top versus bottom fourth of baseline cIMT with incidence of first‐ever carotid plaque. Abbreviations: CI, confidence interval; RR, relative risk; full study names are provided in the footnote of Table 1

The strengths of associations of baseline cIMT with incidence of first‐ever carotid plaque are depicted in Figure 2. Overall, using random‐effects meta‐analysis, the pooled RR for incidence of first‐ever carotid plaque of individuals in the top fourth compared to those in the bottom fourth of baseline cIMT was 1.78 (95% CI: 1.53‐2.07; *P* < .001). The between‐study heterogeneity *I* ^2^ was low at 2.8% (*P* = .404). A sensitivity analysis applying fixed‐effect meta‐analysis yielded a pooled RR of 1.77 (95% CI: 1.54‐2.05; *P* < .001).

**Figure 3:**
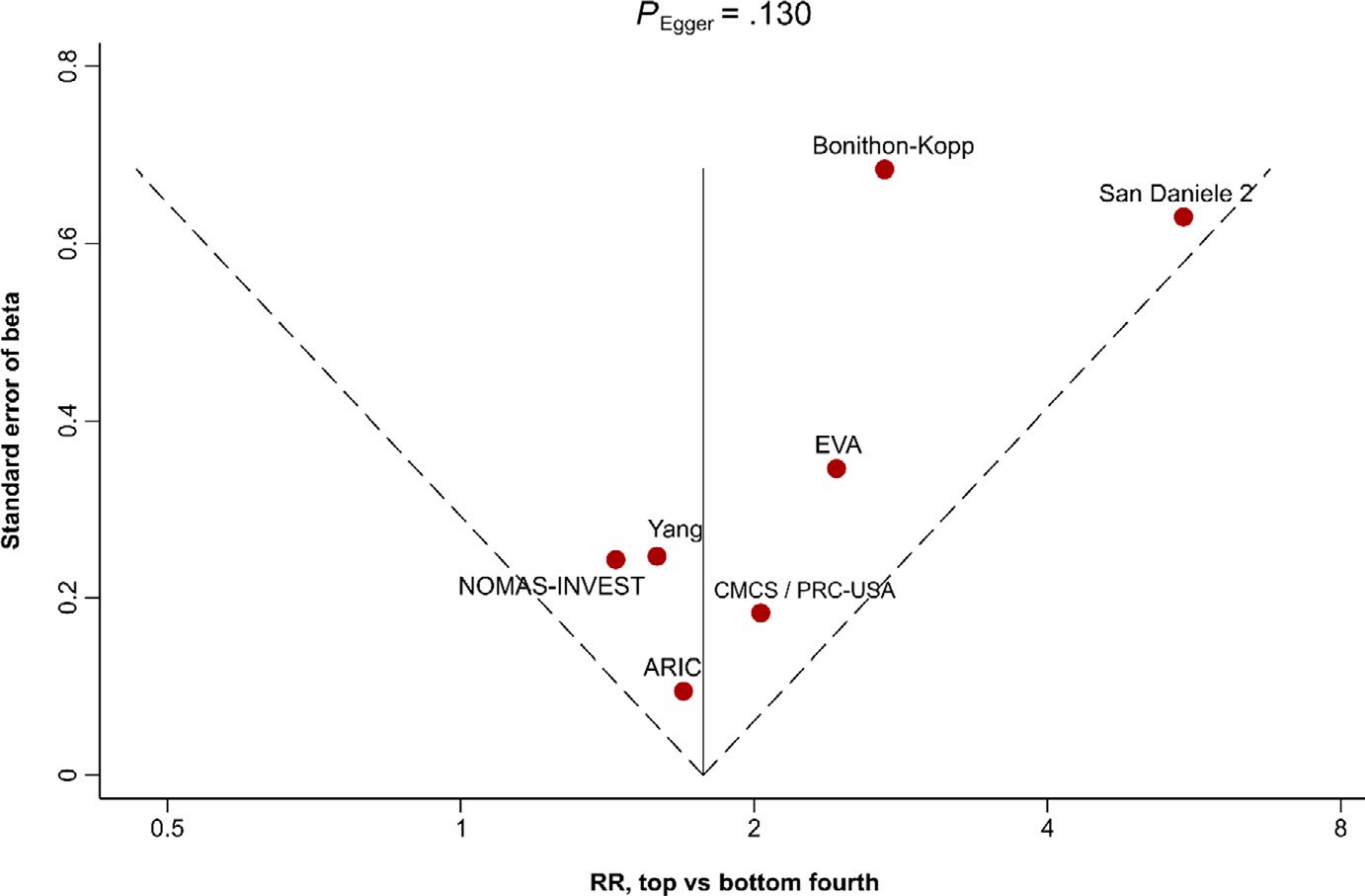
Funnel plot of association of top versus bottom fourth of baseline cIMT with incidence of first‐ever carotid plaque. Abbreviations: RR, relative risk; full study names are provided in the footnote of Table 1

Figure 3 shows the funnel plot presenting the association of baseline cIMT with incidence of first‐ever carotid plaque. Asymmetry of the funnel plot as assessed by Egger's test was not statistically significant (*P* = .130).

### 3.4 Associations with study‐level characteristics

**Figure 4:**
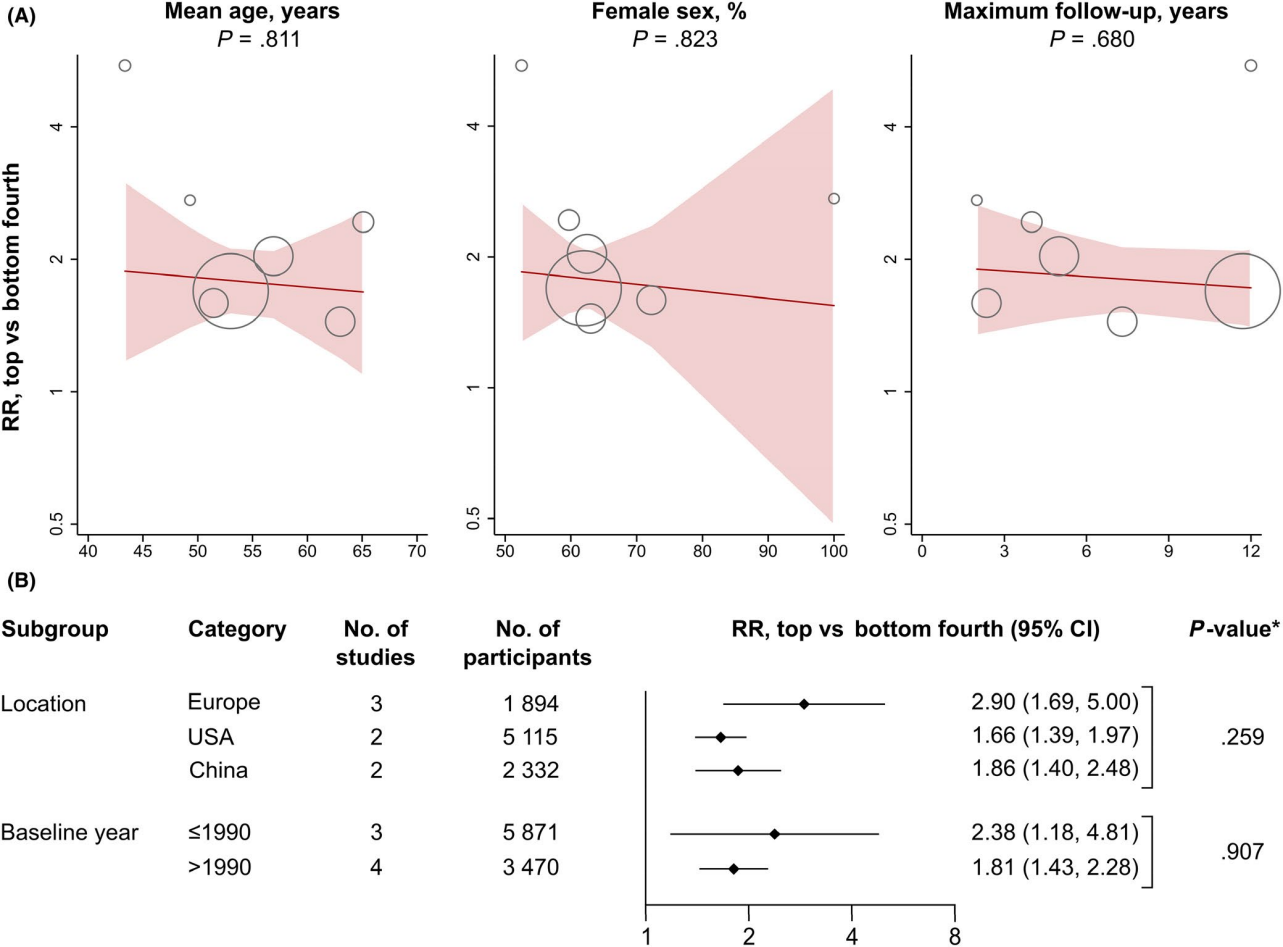
Associations according to study‐level characteristics. A, Associations according to continuous study‐level characteristics. B, Associations according to categorical study‐level characteristics. Abbreviations: CI, confidence interval; RR, relative risk. *P‐values are derived from meta‐regression

Analyses according to continuous and categorical study‐level characteristics are presented in Figure 4. There was no evidence that the strengths of associations differed according to mean baseline age, percentage of female participants, maximum length of follow‐up, geographical location or year of baseline (all *P* > .05).

## 4 DISCUSSION

This literature‐based meta‐analysis of seven prospective studies involving a total of 9341 individuals identified a positive association between increased baseline cIMT and incidence of first‐ever carotid plaque. Individuals in the top fourth compared to those in the bottom fourth of baseline cIMT had, on average, a + 78% higher relative risk for developing first‐ever carotid plaque (95% CI: 53%‐107%). There were differences between the studies' mean baseline ages, percentages of female participants, maximum lengths of follow‐up, geographical locations and years of baseline, but none of them affected the combined RR significantly.

The results of the current analysis are in line with results of two other general population studies, which reported similar findings but were not included in our meta‐analysis because they focused on the number rather than the presence of carotid plaque as an outcome. The Study of Health in Pomerania demonstrated that the risk ratio for a higher number of segments affected by carotid plaque was 1.53 (95% CI: 1.12‐2.07) for subjects in the highest fourth of baseline cIMT measured in the CCA compared to those in the lowest fourth.^39^ Furthermore, the Tromsø study analysed 2610 individuals free of carotid plaque at baseline and reported that an increase in cIMT of one SD yielded, on average, an odds ratio of 1.43 (95% CI: 1.29‐1.58) for risk of developing a higher number of plaques.^40^ In contrast, the population‐based REFINE‐Reykjavik study investigated predictors of new plaque formation in 462 individuals free of carotid plaque at baseline and found no statistically significant association between increased cIMT at the CCA and risk of new plaque formation (RR 1.12 [95% CI: 0.94‐1.33]).^41^

Our results are also comparable to prior studies conducted in individuals at high cardiovascular risk. The Campania Salute Network investigated the association per higher quintile of cIMT with incident carotid plaque in a population consisting of 2143 treated hypertensive patients without baseline carotid plaque and observed a hazard ratio of 1.42 (95% CI: 1.33‐1.52).^42^ A further study of asymptomatic subjects with moderate carotid stenosis identified pathological cIMT, defined as > 1 mm, to be predictive of risk for plaque progression reporting a multivariable‐adjusted odds ratio of 2.28 (95% CI: 1.18‐4.43).^43^ In a longitudinal study of 103 patients with end‐stage renal disease, baseline cIMT correlated positively with the formation rate of new atherosclerotic plaques on univariate analysis, but was not statistically significant in a multiple regression model for new plaque formation.^44^ In a subpopulation of the NEFRONA study involving 1553 participants with different stages of chronic kidney disease, cIMT was associated with new plaque progression in dialysis patients.^45^ In another subpopulation of the NEFRONA study including 237 patients undergoing peritoneal dialysis, individuals in the median and highest third of baseline cIMT had a higher risk for progression of atherosclerotic disease compared to individuals in the lowest third of baseline cIMT.^46^

In order to predict the genesis of carotid plaques, it is important to study initiation and progression of atherosclerosis. The mechanisms behind the development of atherosclerosis consist of many different facets including inflammation, endothelial dysfunction, oxidation, lipid accumulation, proliferation and migration of vascular smooth muscle cells, which lead to manifestation of atherosclerotic plaques in the vessel walls that can further cause thrombosis and stenosis in human arteries.^1^ Intima‐media thickness is therefore believed to serve as early marker of atherogenic vessel wall remodelling. However, this theory has recently been challenged. Conventional B‐mode ultrasound techniques cannot distinguish precisely the intimal layer from the medial layer, and it has been shown that intima‐media thickness comprises a significant portion of the tunica media and only a little part of the tunica intima (approximately 80% versus 20%).^47^ An advanced stage of atherosclerosis comprises the formation of atherosclerotic plaques, which is considered to be mainly a process of the intimal layer of arteries.^48^, ^49^ Therefore, it has been suspected that increased intima‐media thickness does not necessarily reflect early stages of atherosclerosis, but it rather mirrors an arterial hypertrophic response to high blood pressure.^12^ Additionally, it has been discussed that alterations of the blood vessels involve a variety of haemodynamic factors such as wall shear stress or tensile stress, which could also lead to arterial wall thickening.^50^ In this case, cIMT would not appropriately reflect the development and progression of carotid plaque. Despite these methodological challenges, our analysis convincingly demonstrated that elevated baseline cIMT was associated with incidence of first‐ever carotid plaque.

Several clinical guidelines state that ultrasound‐based assessment of carotid plaque may be considered in order to identify individuals at high risk for developing clinically manifest CVD.^51^, ^52^, ^53^ Our present report shows that baseline cIMT and the risk of incident carotid plaque are closely intertwined. By extension, cIMT—provided it is measured at high quality—may be useful to identify high‐risk individuals at an even earlier stage of the atherosclerosis development. A previous evaluation of the added predictive value of cIMT assessment on top of conventional risk factors has suggested modest improvements in risk discrimination and risk reclassification.^54^ While this evaluation focused on cIMT assessments at a single point in time, repeat assessments taking into account person‐based cIMT trajectories might afford an even greater benefit.

Although a significant association between increased baseline cIMT and first‐ever carotid plaque has been obtained, this does not necessarily imply a direct relationship between them. Elevated cIMT and carotid plaque have both been associated with a number of cardiovascular risk factors^13^, ^55^, ^56^, ^57^, ^58^, ^59^ and also share a considerable amount of these risk factors. Hence, the possibility of a confounding factor driving the current result towards positivity exists. However, most of the studies involved in the analysis have adjusted for the majority of traditional cardiovascular risk factors including sex, age, smoking, hypertension, diabetes mellitus, hypercholesterolaemia and history of CVD. Multivariate adjustment including prominent cardiovascular risk factors minimises the possibility of confounding. Nevertheless, the probable involvement of an unknown third factor driving the result remains. Since the current results are based on published material only, it was not possible to define a uniform model adjusting for the same risk factors for every study. The approach of individual‐participant‐data meta‐analysis would enable harmonisation of adjustments across studies and consequently increase comparability, which would lead to more representational results and conclusions.

Besides varying levels of adjustment, differences in measurement techniques of cIMT and carotid plaque could hamper the comparison between individual studies.^5^ cIMT can be measured on different sites of the carotid artery, at the CCA, the carotid bifurcation, and the internal carotid artery, at the left and right side of the neck, and at the near and far wall of the carotid artery. Furthermore, different metrics can be applied to compute summary measures representing cIMT. Multiple cIMT measures can, for example, be averaged to obtain mean cIMT, the maximum value of single measurements can be computed to get maximum cIMT, or several maximum values can be averaged to obtain mean‐maximum cIMT. In the current analysis, cIMT definitions varied between the studies in terms of measurement sides (left side and/or right side), measurement walls (far wall and/or near wall) and measurement metric (mean cIMT or mean‐maximum cIMT). A positive aspect is that all results were based on measurements at the CCA and most studies measured cIMT at the far wall. This is most likely the case because it is currently recommended that cIMT should preferably be measured at the far wall of the CCA, as this measurement method is anatomically validated.^2^, ^60^, ^61^ In addition, reproducibility of cIMT at the CCA has been identified to be superior to reproducibility at the internal carotid artery and the carotid bulb.^62^ Definition of carotid plaque also varied between contributing studies. The Mannheim Carotid Intima‐Media Thickness and Plaque Consensus defined plaques as “focal structures encroaching into the arterial lumen of at least 0.5 mm or 50% of the surrounding” or as intima‐media thickness > 1.5 mm.^60^ In the current analysis, studies defined carotid plaque based on several criteria including focal plaque shape, increased cIMT and arterial wall texture. Although considerable differences in assessment of cIMT and carotid plaque were obtained, between‐study heterogeneity obtained with the *I* ^2^ statistic was rather low with 2.8%. Nevertheless, differences in characterisation of cIMT and carotid plaque limit the comparability between single studies. Consequently, harmonisation of measurement protocols would be a valuable approach.

Considerable strengths of the current report include the comprehensive systematic literature review and the harmonisation of different scales of RRs. One limitation of the current analysis lies in the differences of cIMT and carotid plaque assessment. Furthermore, all studies involved participants of the general population. Hence, results might not be applicable to other populations (eg individuals at high cardiovascular risk). In addition, a significant weakness is the reliance on published information rather than original data. The use of literature‐based results did not allow to investigate the effect of interim CVD events, which could have a potential impact on development of future carotid plaque due to initiation or modification of treatments or due to changes in lifestyle. An individual‐participant‐data meta‐analysis would enable consistent strategies in data analysis and would thereby enhance comparability between studies.

In conclusion, in general population studies increased baseline cIMT is associated with development of future carotid plaque in individuals free of carotid plaque at baseline.

## CONFLICT OF INTEREST

LT reports a grant from the Dr. Johannes and Hertha Tuba Foundation and nonfinancial support from Sanofi outside the submitted work. GK reports nonfinancial support from Sanofi and Pfizer outside the submitted work. LS reports nonfinancial support from Sanofi outside the submitted work. PW reports grants from the Austrian Science Fund (FWF) [P 32488] and the Dr. Johannes and Hertha Tuba Foundation, personal fees from Novartis Pharmaceuticals and nonfinancial support from Bayer, Daiichi Sankyo and Sanofi outside the submitted work.

## AUTHOR CONTRIBUTIONS

LT, GK and PW conducted the systematic literature search, analysed data and wrote the manuscript. LS revised the manuscript for intellectual content. PW is the guarantor of this work and, as such, had full access to all the data in the study and takes responsibility for the integrity of the data and the accuracy of the data analysis.
